# 3-[2-(1*H*-Benzimidazol-2-ylsulfan­yl)eth­yl]-1,3-oxazolidin-2-one

**DOI:** 10.1107/S1600536810045897

**Published:** 2010-11-13

**Authors:** Ahmed Moussaif, El Mokhtar Essassi, Said Lazar, Hafid Zouihri, Jean Michel Leger

**Affiliations:** a Centre National de l’Energie, des Sciences et des Techniques Nucléaires, Maamoura Kenitra, Morocco; b Institut of Nanomaterials and Nanotechnology, INANOTECH, Avenue de l Armée, Royale, Rabat, Morocco; c Laboratoire de Biochimie, Environnement et Agroalimentaire (URAC 36), Faculté des Sciences et Techniques Mohammedia, Université Hassan II, Mohammedia-Casablana, BP 146, 20800 Mohammedia, Morocco; d Laboratoires de Diffraction des Rayons X, Centre Nationale pour la Recherche Scientifique et Technique, Rabat, Morocco; e Laboratoire de Chimie Physique et Minérale, Service de Cristallographie, Université Victor Segalen Bordeaux II, France

## Abstract

In the title compound, C_12_H_13_N_3_O_2_S, the oxazolidin ring displays an envelope conformation. The dihedral angle between the benzimidazole ring and the 1,3-oxazolidin-2-one mean plane is 69.85 (13)°. In the crystal, mol­ecules are linked by inter­molecular N—H⋯N hydrogen bonds, forming a chain parallel to the *b* axis.

## Related literature

For the structures of oxazolidin-2-one linked to dioxoindolin, quinoxaline, benzodiazepin-2(3*H*)-one and indolo[2,3-*b*]quinoxalin, see: Al Subari *et al.* (2010*a* ▶,*b* ▶); Ahoya *et al.* (2010 ▶); Ballo *et al.* (2010 ▶). For puckering parameters, see: Cremer & Pople (1975 ▶).
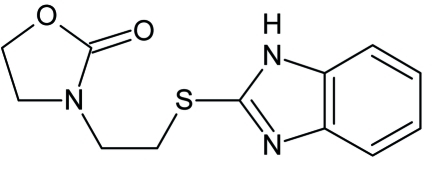


## Experimental

### 

#### Crystal data

C_12_H_13_N_3_O_2_S
*M* *_r_* = 263.31
Orthorhombic, 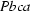

*a* = 8.258 (1) Å
*b* = 10.074 (1) Å
*c* = 29.201 (3) Å
*V* = 2429.3 (5) Å^3^
*Z* = 8
Cu *K*α radiation
μ = 2.37 mm^−1^
*T* = 296 K
0.25 × 0.10 × 0.05 mm

#### Data collection

Enraf–Nonius CAD-4 diffractometer
Absorption correction: ψ scan (North *et al.*, 1968 ▶) *T*_min_ = 0.589, *T*_max_ = 0.891
2065 measured reflections
2065 independent reflections
1580 reflections with *I* > 2σ(*I*)
2 standard reflections every 90 min  intensity decay: none

#### Refinement

*R*[*F* ^2^ > 2σ(*F* ^2^)] = 0.048
*wR*(*F* ^2^) = 0.138
*S* = 1.04
2065 reflections
164 parameters
H-atom parameters constrained
Δρ_max_ = 0.50 e Å^−3^
Δρ_min_ = −0.26 e Å^−3^

### 

Data collection: *CAD-4 Software* (Enraf–Nonius, 1989 ▶); cell refinement: *CAD-4 Software*; data reduction: *CAD-4 Software*; program(s) used to solve structure: *SHELXS97* (Sheldrick, 2008 ▶); program(s) used to refine structure: *SHELXL97* (Sheldrick, 2008 ▶); molecular graphics: *ORTEPIII* (Burnett & Johnson, 1996 ▶), *ORTEP-3 for Windows* (Farrugia, 1997 ▶) and *PLATON* (Spek, 2009 ▶); software used to prepare material for publication: *publCIF* (Westrip, 2010 ▶).

## Supplementary Material

Crystal structure: contains datablocks I, global. DOI: 10.1107/S1600536810045897/dn2620sup1.cif

Structure factors: contains datablocks I. DOI: 10.1107/S1600536810045897/dn2620Isup2.hkl

Additional supplementary materials:  crystallographic information; 3D view; checkCIF report

## Figures and Tables

**Table 1:** Hydrogen-bond geometry (Å, °)

*D*—H⋯*A*	*D*—H	H⋯*A*	*D*⋯*A*	*D*—H⋯*A*
N9—H9⋯N7^i^	0.86	2.03	2.866 (3)	165

Symmetry code: (i) 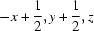.

**Fig. 1.**
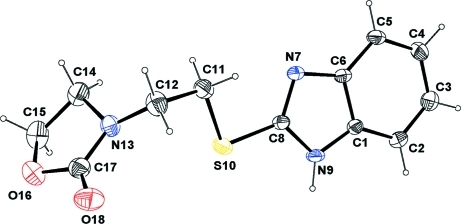
Molecular view of the title compound showing the atom-labeling scheme. Displacement ellipsoids are drawn at the 30% probability level. H atoms are represented as small spheres of arbitrary radii.

**Fig. 2.**
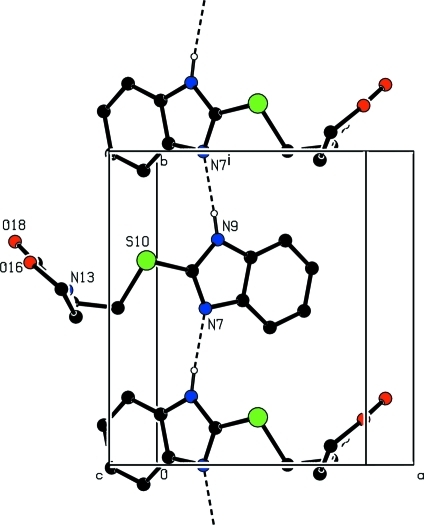
Partial packing view showing the chain formed by N-H···N hydrogen bondings. H atoms not involved in hydrogen bonds have been omitted for clarity. [Symmetry code: (i) -x+1/2, y+1/2, z ]

## supplementary crystallographic information

### Comment

The synthesis of new oxindole derivatives having an oxazolindin-2-one unit has been detailed in recent reports (Al Subari *et al.*, 2010*a*,*b*; Ahoya *et al.*, 2010; Ballo *et al.*,2010).

In the new oxazolidin-2-one, C_12_H_13_N_3_O_2_S, the dihedral angle between the 1H-benzimidazole ring and the 1,3-oxazolidin-2-one mean plane is: 69.85 (13)° (Fig.1). The oxazolidin ring is not planar but display envelope conformation on C14 with puckering parameters Q(2) = 0.258 (3) Å and φ(2) = 63.3 (7) ° (Cremer & Pople, 1975).

In the crystal structure, the molecules are linked by intermolecular N—H···N hydrogen bonds forming a chain parallel to the *b* axis (Table 1, Fig. 2).

### Experimental

To the solution of benzimidazole-2-thione (1,35 g, 9 mmoles) and dichloroethyl amine hydrochloride (2,41 g, 13.5 mmoles) in dimethylformamide (80 ml) were added potassium carbonate (4,14 g, 30 mmoles) and tetra-*n*-butylammonium bromide (0,10 g, 0,3 mmoles). The resulting mixture was refluxed for 4 h. After filtering the solvent was removed and the residue was purified by column chromatography on silica gel (Hexane/AcOEt: 60/40) to afford the title compound.

Yield = 55%

F = 230–232 °C (ethanol-water).

RMN ^1^H (d p.p.m.): 3.57: SCH_2_ (2*H*, t, *J* = 6.25 Hz); 3,36: NCH_2_ (4*H*, m); 4.23: OCH_2_ (4*H*, t, *J* = 6,25 Hz); 7.30–7.70: CH (benzénique)(8*H*, m); 11.57: NH (1*H*, s)

Mass Spectre IE: *M*^+^ (m/*z*=263).

### Refinement

All H atoms were fixed geometrically and treated as riding with C—H = 0.97 Å (methyne) and 0.93Å (aromatic) with *U*_iso_(H) = 1.2Ueq(C).

### Crystal data

**Table:**

C_12_H_13_N_3_O_2_S	*F*(000) = 1104
*M**_r_* = 263.31	*D*_x_ = 1.440 Mg m^−^^3^
Orthorhombic, *P**b**c**a*	Cu *K*α radiation, λ = 1.54180 Å
Hall symbol: -P 2ac 2ab	Cell parameters from 25 reflections
*a* = 8.258 (1) Å	θ = 25–35°
*b* = 10.074 (1) Å	µ = 2.37 mm^−^^1^
*c* = 29.201 (3) Å	*T* = 296 K
*V* = 2429.3 (5) Å^3^	Plate, colourless
*Z* = 8	0.25 × 0.10 × 0.05 mm

### Data collection

**Table:**

Enraf–Nonius CAD-4 diffractometer	1580 reflections with *I* > 2σ(*I*)
Radiation source: fine-focus sealed tube	*R*_int_ = 0.0000
graphite	θ_max_ = 64.9°, θ_min_ = 3.0°
ω–2θ scans	*h* = 0→9
Absorption correction: ψ scan (North *et al.*, 1968)	*k* = 0→11
*T*_min_ = 0.589, *T*_max_ = 0.891	*l* = 0→34
2065 measured reflections	2 standard reflections every 90 min
2065 independent reflections	intensity decay: none

### Refinement

**Table:**

Refinement on *F*^2^	Secondary atom site location: difference Fourier map
Least-squares matrix: full	Hydrogen site location: inferred from neighbouring sites
*R*[*F*^2^ > 2σ(*F*^2^)] = 0.048	H-atom parameters constrained
*wR*(*F*^2^) = 0.138	*w* = 1/[σ^2^(*F*_o_^2^) + (0.0753*P*)^2^ + 1.285*P*] where *P* = (*F*_o_^2^ + 2*F*_c_^2^)/3
*S* = 1.04	(Δ/σ)_max_ < 0.001
2065 reflections	Δρ_max_ = 0.50 e Å^−^^3^
164 parameters	Δρ_min_ = −0.26 e Å^−^^3^
0 restraints	Extinction correction: *SHELXL97* (Sheldrick, 2008), Fc^*^=kFc[1+0.001xFc^2^λ^3^/sin(2θ)]^-1/4^
Primary atom site location: structure-invariant direct methods	Extinction coefficient: 0.0023 (3)

### Special details

**Table:**

Geometry. All e.s.d.'s (except the e.s.d. in the dihedral angle between two l.s. planes) are estimated using the full covariance matrix. The cell e.s.d.'s are taken into account individually in the estimation of e.s.d.'s in distances, angles and torsion angles; correlations between e.s.d.'s in cell parameters are only used when they are defined by crystal symmetry. An approximate (isotropic) treatment of cell e.s.d.'s is used for estimating e.s.d.'s involving l.s. planes.
Refinement. Refinement of *F*^2^ against ALL reflections. The weighted *R*-factor *wR* and goodness of fit *S* are based on *F*^2^, conventional *R*-factors *R* are based on *F*, with *F* set to zero for negative *F*^2^. The threshold expression of *F*^2^ > σ(*F*^2^) is used only for calculating *R*-factors(gt) *etc*. and is not relevant to the choice of reflections for refinement. *R*-factors based on *F*^2^ are statistically about twice as large as those based on *F*, and *R*- factors based on ALL data will be even larger.

### Fractional atomic coordinates and isotropic or equivalent isotropic displacement parameters (Å^2^)

**Table:**

	*x*	*y*	*z*	*U*_iso_*/*U*_eq_	
C1	0.4252 (3)	0.6613 (3)	0.32287 (8)	0.0308 (6)	
C2	0.5563 (4)	0.7150 (3)	0.29974 (10)	0.0418 (7)	
H2	0.5765	0.8058	0.2996	0.050*	
C3	0.6553 (4)	0.6266 (3)	0.27685 (11)	0.0491 (8)	
H3	0.7451	0.6589	0.2612	0.059*	
C4	0.6247 (4)	0.4910 (3)	0.27658 (10)	0.0452 (8)	
H4	0.6930	0.4349	0.2602	0.054*	
C5	0.4960 (4)	0.4382 (3)	0.29994 (10)	0.0387 (7)	
H5	0.4772	0.3471	0.3000	0.046*	
C6	0.3939 (3)	0.5243 (3)	0.32364 (8)	0.0300 (6)	
N7	0.2541 (3)	0.4990 (2)	0.34878 (7)	0.0331 (5)	
C8	0.2044 (3)	0.6179 (3)	0.36218 (9)	0.0323 (6)	
N9	0.3018 (3)	0.7182 (2)	0.34785 (7)	0.0341 (5)	
H9	0.2890	0.8014	0.3533	0.041*	
S10	0.03324 (10)	0.65185 (8)	0.39498 (3)	0.0466 (3)	
C11	−0.0806 (4)	0.5004 (3)	0.38524 (11)	0.0461 (8)	
H11A	−0.0786	0.4786	0.3529	0.055*	
H11B	−0.0306	0.4279	0.4019	0.055*	
C12	−0.2545 (4)	0.5167 (4)	0.40086 (11)	0.0515 (8)	
H12A	−0.3071	0.5824	0.3817	0.062*	
H12B	−0.3109	0.4331	0.3967	0.062*	
N13	−0.2688 (3)	0.5569 (3)	0.44810 (8)	0.0421 (6)	
C14	−0.2251 (4)	0.4720 (4)	0.48622 (11)	0.0524 (9)	
H14A	−0.1101	0.4527	0.4866	0.063*	
H14B	−0.2857	0.3895	0.4859	0.063*	
C15	−0.2743 (4)	0.5603 (4)	0.52586 (12)	0.0624 (10)	
H15A	−0.3162	0.5079	0.5511	0.075*	
H15B	−0.1832	0.6124	0.5366	0.075*	
O16	−0.3993 (3)	0.6452 (2)	0.50692 (8)	0.0568 (6)	
C17	−0.3857 (4)	0.6431 (3)	0.46087 (11)	0.0465 (8)	
O18	−0.4713 (3)	0.7117 (3)	0.43673 (9)	0.0683 (8)	

### Atomic displacement parameters (Å^2^)

**Table:**

	*U*^11^	*U*^22^	*U*^33^	*U*^12^	*U*^13^	*U*^23^
C1	0.0367 (14)	0.0282 (15)	0.0275 (12)	0.0002 (11)	−0.0008 (11)	0.0017 (10)
C2	0.0495 (18)	0.0290 (15)	0.0469 (17)	−0.0053 (13)	0.0046 (14)	0.0035 (12)
C3	0.0486 (18)	0.0473 (18)	0.0513 (18)	−0.0047 (16)	0.0098 (15)	0.0036 (15)
C4	0.0474 (17)	0.0447 (18)	0.0436 (17)	0.0103 (15)	0.0090 (13)	−0.0055 (13)
C5	0.0432 (16)	0.0254 (14)	0.0474 (16)	0.0052 (13)	0.0044 (13)	−0.0010 (12)
C6	0.0351 (14)	0.0218 (13)	0.0330 (13)	0.0008 (11)	−0.0046 (11)	0.0008 (10)
N7	0.0381 (12)	0.0221 (12)	0.0391 (12)	0.0007 (9)	0.0051 (10)	0.0001 (9)
C8	0.0376 (15)	0.0227 (13)	0.0365 (14)	0.0019 (11)	0.0012 (11)	−0.0006 (11)
N9	0.0406 (13)	0.0176 (11)	0.0441 (13)	−0.0002 (10)	0.0036 (10)	−0.0018 (9)
S10	0.0457 (5)	0.0336 (4)	0.0604 (5)	−0.0002 (3)	0.0160 (3)	−0.0094 (3)
C11	0.0460 (17)	0.0395 (18)	0.0527 (18)	−0.0037 (14)	0.0117 (15)	−0.0059 (13)
C12	0.0413 (16)	0.061 (2)	0.0521 (18)	−0.0089 (16)	0.0026 (15)	−0.0091 (16)
N13	0.0346 (13)	0.0480 (15)	0.0437 (13)	0.0020 (12)	0.0031 (11)	0.0000 (11)
C14	0.0382 (17)	0.059 (2)	0.060 (2)	0.0090 (16)	0.0014 (15)	0.0143 (17)
C15	0.047 (2)	0.088 (3)	0.053 (2)	−0.003 (2)	−0.0054 (15)	0.0077 (19)
O16	0.0518 (14)	0.0638 (17)	0.0549 (13)	0.0065 (12)	0.0019 (11)	−0.0115 (11)
C17	0.0414 (17)	0.0403 (17)	0.0578 (19)	−0.0041 (15)	0.0020 (15)	−0.0040 (15)
O18	0.0727 (17)	0.0523 (16)	0.0798 (18)	0.0179 (14)	−0.0127 (14)	0.0047 (13)

### Geometric parameters (Å, °)

**Table:**

S10—C8	1.741 (3)	C4—C5	1.370 (4)
S10—C11	1.815 (3)	C5—C6	1.394 (4)
O16—C15	1.450 (4)	C11—C12	1.516 (5)
O16—C17	1.350 (4)	C14—C15	1.515 (5)
O18—C17	1.214 (4)	C2—H2	0.9298
N7—C6	1.392 (3)	C3—H3	0.9299
N7—C8	1.325 (4)	C4—H4	0.9308
N9—C1	1.378 (3)	C5—H5	0.9308
N9—C8	1.358 (4)	C11—H11A	0.9697
N13—C12	1.443 (4)	C11—H11B	0.9698
N13—C14	1.449 (4)	C12—H12A	0.9694
N13—C17	1.351 (4)	C12—H12B	0.9700
N9—H9	0.8597	C14—H14A	0.9694
C1—C2	1.386 (4)	C14—H14B	0.9702
C1—C6	1.404 (4)	C15—H15A	0.9704
C2—C3	1.381 (4)	C15—H15B	0.9694
C3—C4	1.389 (4)		
			
C8—S10—C11	99.74 (15)	C1—C2—H2	121.75
C15—O16—C17	108.2 (2)	C3—C2—H2	121.73
C6—N7—C8	104.3 (2)	C2—C3—H3	118.96
C1—N9—C8	107.0 (2)	C4—C3—H3	119.10
C12—N13—C14	123.3 (3)	C3—C4—H4	119.32
C12—N13—C17	120.2 (3)	C5—C4—H4	119.35
C14—N13—C17	110.2 (2)	C4—C5—H5	120.87
C8—N9—H9	126.57	C6—C5—H5	120.79
C1—N9—H9	126.47	S10—C11—H11A	109.53
N9—C1—C6	105.3 (2)	S10—C11—H11B	109.51
N9—C1—C2	132.3 (3)	C12—C11—H11A	109.50
C2—C1—C6	122.4 (3)	C12—C11—H11B	109.51
C1—C2—C3	116.5 (3)	H11A—C11—H11B	108.10
C2—C3—C4	121.9 (3)	N13—C12—H12A	108.88
C3—C4—C5	121.3 (3)	N13—C12—H12B	108.92
C4—C5—C6	118.3 (3)	C11—C12—H12A	108.95
N7—C6—C5	130.5 (3)	C11—C12—H12B	108.85
C1—C6—C5	119.5 (2)	H12A—C12—H12B	107.78
N7—C6—C1	109.9 (2)	N13—C14—H14A	111.78
N7—C8—N9	113.5 (2)	N13—C14—H14B	111.70
S10—C8—N7	126.3 (2)	C15—C14—H14A	111.82
S10—C8—N9	120.3 (2)	C15—C14—H14B	111.83
S10—C11—C12	110.6 (2)	H14A—C14—H14B	109.49
N13—C12—C11	113.3 (3)	O16—C15—H15A	110.90
N13—C14—C15	100.0 (3)	O16—C15—H15B	110.88
O16—C15—C14	104.2 (3)	C14—C15—H15A	110.88
O18—C17—N13	128.4 (3)	C14—C15—H15B	110.91
O16—C17—O18	121.4 (3)	H15A—C15—H15B	109.00
O16—C17—N13	110.2 (3)		
			
C11—S10—C8—N7	−20.2 (3)	C14—N13—C17—O16	14.2 (4)
C11—S10—C8—N9	159.9 (2)	C14—N13—C12—C11	−68.2 (4)
C8—S10—C11—C12	−166.3 (2)	C12—N13—C17—O16	167.1 (3)
C15—O16—C17—O18	−176.2 (3)	C12—N13—C17—O18	−12.7 (5)
C15—O16—C17—N13	4.0 (3)	N9—C1—C6—N7	−0.1 (3)
C17—O16—C15—C14	−19.3 (3)	N9—C1—C2—C3	−178.2 (3)
C6—N7—C8—S10	179.8 (2)	N9—C1—C6—C5	178.2 (2)
C8—N7—C6—C1	0.3 (3)	C6—C1—C2—C3	0.5 (4)
C6—N7—C8—N9	−0.3 (3)	C2—C1—C6—N7	−179.2 (2)
C8—N7—C6—C5	−177.8 (3)	C2—C1—C6—C5	−0.9 (4)
C1—N9—C8—N7	0.3 (3)	C1—C2—C3—C4	0.6 (5)
C1—N9—C8—S10	−179.83 (18)	C2—C3—C4—C5	−1.4 (5)
C8—N9—C1—C6	−0.1 (3)	C3—C4—C5—C6	1.0 (5)
C8—N9—C1—C2	178.9 (3)	C4—C5—C6—C1	0.1 (4)
C12—N13—C14—C15	−176.6 (3)	C4—C5—C6—N7	178.0 (3)
C14—N13—C17—O18	−165.6 (3)	S10—C11—C12—N13	−55.8 (4)
C17—N13—C12—C11	142.6 (3)	N13—C14—C15—O16	25.6 (3)
C17—N13—C14—C15	−24.7 (3)		

### Hydrogen-bond geometry (Å, °)

**Table:**

*D*—H···*A*	*D*—H	H···*A*	*D*···*A*	*D*—H···*A*
N9—H9···N7^i^	0.86	2.03	2.866 (3)	165

Symmetry codes: (i) −*x*+1/2, *y*+1/2, *z*.
